# i*Neuron‐GEM*: Identifying Metabolic Alterations in Neuronal Subtypes Associated with Alzheimer's Disease Pathophysiology

**DOI:** 10.1002/alz70855_105320

**Published:** 2025-12-24

**Authors:** Boyu Jiang, Anke Marije Tukker, Hyunjin Kim, Aaron B Bowman, Priyanka Baloni

**Affiliations:** ^1^ Purdue University, West Lafayette, IN, USA

## Abstract

**Background:**

Metabolic dysfunctions have been increasingly studied in neurodegenerative diseases like Alzheimer's Disease (AD), where neurons must meet high energy demands for neurotransmission and synaptic activity through oxidative phosphorylation and glycolysis‐driven ATP production. Metabolic disturbances in these functions were reported for pathophysiological changes in AD. Detailed mechanisms underlying metabolic alterations in specific neuronal subtypes remain insufficiently explored.

**Method:**

We generated a neuronal subtype‐specific genome‐scale metabolic network – i*Neuron‐GEM*, using single‐nucleus RNA‐sequencing (snRNA‐seq) data from two AD cohort studies: the Religious Orders Study and Memory and Aging Project (ROSMAP) and Seattle Alzheimer's Disease Brain Cell Atlas (SEA‐AD). We tested the accuracy of i*Neuron‐GEM* reconstruction using neuron‐specific metabolic tasks and refined the reconstruction with metabolomics data from human induced pluripotent stem cell (iPSC)‐derived neurons and cerebrospinal fluid. Using i*Neuron‐GEM*, we performed *in silico* metabolic analysis on snRNA‐seq data from the Mount Sinai Neuropsychiatric Symptoms in AD (NPS‐AD) Study to identify key metabolic changes in neuronal subtypes. We also improved our metabolic predictions using supervised machine learning approaches to identify disrupted metabolic reactions and pathways in inhibitory and excitatory neurons associated with AD pathology.

**Result:**

We report the first *in silico* metabolic reconstruction of neurons, i*Neuron‐GEM*, containing 1339 metabolites, 3900 metabolic reactions, and 1703 genes. This metabolic reconstruction can achieve essential neuronal metabolic functions. Results from integrated metabolic analysis combined with a supervised machine learning approach identified that excitatory neurons exhibited more significant metabolic alterations between healthy and AD individuals compared to inhibitory neurons. These alterations included metabolic reactions linked to genes (i.e. *ACSL1, ELOVL2*, and *ACSBG2*) and metabolites (i.e. homoserine, ornithine, and choline), concordant with those implicated in AD progression. Our analysis revealed key mechanistic changes in fatty acid synthesis, glycerophospholipid metabolism and branched‐chain amino acid catabolism in excitatory neurons during AD progression.

**Conclusion:**

We present the first manually curated metabolic reconstruction of human neurons, named i*Neuron‐GEM*. This metabolic reconstruction is useful for integrating multi‐omics data and predicting metabolic changes in neuronal subtypes in AD. The findings from integrated i*Neuron‐GEM* analyses enable the identification of metabolic signatures and druggable targets in AD.

